# Variations in Tongue-Palate Swallowing Pressures When Swallowing Xanthan Gum-Thickened Liquids

**DOI:** 10.1007/s00455-014-9561-6

**Published:** 2014-08-03

**Authors:** Catriona M. Steele, Sonja M. Molfenter, Melanie Péladeau-Pigeon, Rebecca C. Polacco, Clemence Yee

**Affiliations:** 1 Toronto Rehabilitation Institute – University Health Network, 550 University Avenue, #12-101, Toronto, ON M5G 2A2 Canada; 2 University of Toronto, Toronto, ON Canada; 3 Bloorview Research Institute, Toronto, ON Canada; 4 New York University, New York, NY USA

**Keywords:** Deglutition, Deglutition disorders, Swallowing, Tongue, Tongue pressure, Viscosity

## Abstract

Thickened liquids are frequently recommended to reduce the risk of aspiration in patients with oropharyngeal dysphagia. Although it has previously been reported that tongue-palate pressures increase when swallowing spoon-thick and semi-solid consistencies compared to thin liquids, relatively little is known about how swallowing behaviors differ when swallowing liquids of nectar- or honey-thick consistency. Furthermore, previous studies have primarily used starch-based thickeners, and little is known about swallowing behaviors with xanthan gum-thickened liquids, which have recently been introduced for dysphagia management. In this study, we measured variations in tongue-palate pressures during the swallowing of liquids thickened to apparent viscosities of 190, 250, and 380 mPa s at 50/s using increasing concentrations of xanthan gum (0.5, 0.63 and 0.87 w/w%). The viscosity differences between these nectar- and honey-thick stimuli were confirmed to exceed sensory perceptual discrimination thresholds. Data were collected from 78 healthy adults in two sex-balanced age-groups (young; mature) and compared to reference values obtained during water swallowing. The results confirm that increased amplitudes of tongue-palate pressure were used when swallowing the thickened liquid stimuli, compared to swallows of water, and for the honey-thick liquid compared to the two nectar-thick liquids. Age-related reductions were seen in tongue strength but not in swallowing pressures, which fell below 40 % of maximum isometric pressure values. Thus, the use of xanthan gum-thickened liquids is unlikely to tax the swallowing system in terms of tongue pressure generation requirements, even in seniors with reduced maximum isometric tongue pressure measures.

## Introduction: Modulation of Tongue-Palate Pressures in Liquid Swallowing

During the oral phase of swallowing, liquid boluses are initially held in a chamber along the midline groove of the tongue [1]. The tongue moves upwards and forwards, compressing the bolus against the palate and squeezing it backwards in a conveyer-belt like fashion [2]. As the bolus reaches the pharynx, the tongue withdraws from the palate, and sweeps downwards and backwards. This caudally directed sweeping movement facilitates pharyngeal lumen constriction behind the bolus and aids bolus clearance [3]. Given that liquids flow when shear forces are applied [4–6], the pressure gradient arising from bolus compression between the tongue and palate is thought to be a key element in influencing the flow of liquids through the oropharynx [7–10]. Thickened liquids are frequently used as an intervention to compensate for poor oral control and aspiration with thin liquids [11, 12]. However, there is a paucity of evidence regarding effective increments of viscosity to use when thickening liquids for clinical use [13]. It is, therefore, of great importance to develop a better understanding of the mechanisms underlying the flow of liquids through the oropharynx in swallowing.

Previous studies report that the amplitudes of tongue-palate pressure vary when swallowing different consistencies. Lower pressure amplitudes are seen for thin liquids (water, barium) versus semisolids (applesauce, pudding or mashed potato [10, 14]), and for thin liquids compared to nectar- and honey-thick liquids prepared using modified corn-starch thickening agents [15–17]. Sex differences also appear to exist in both maximum isometric and swallowing pressure measures, with females using a greater percentage of their maximum pressure capacity when swallowing, although the literature is equivocal on this point [17–20]. The temporal aspects of tongue-palate pressure application also seem to vary according to bolus flow characteristics: specifically, healthy young adults have been observed to release tongue pressure more gradually for thin liquids compared to nectar-thick liquids [15]. This finding has been interpreted as possibly suggesting longer, more active control of the faster flowing bolus as it enters the pharynx [15].

Xanthan gum-based thickeners are a relatively recent introduction to the dysphagia thickener market. Xanthan gum-thickened liquids are known to have non-Newtonian shear-thinning flow properties, and are amylase-resistant, which is argued to provide greater stability in viscosity during the oral phase of swallowing by preventing bolus dilution with saliva [21, 22]. Additionally, xanthan gum-thickened liquids are described to have low yield-stress (i.e., flow is easily initiated with the application of relatively little force) and to be slippery and have high cohesiveness [6, 23]. They have been reported to lead to less post-swallow residue in the pharynx compared to corn-starch-thickened liquids [24]. However, it remains unknown whether xanthan gum-thickened liquids require increased amplitudes of tongue-palate pressure for bolus propulsion, and whether the temporal profile of tongue-palate pressures vary, compared to those seen with thin liquids. The purpose of this study was to fill this gap in knowledge by measuring tongue-palate pressures during the swallowing of three levels of xanthan gum-thickened liquids compared to water.

## Materials and Methods

### Participants

The sample comprised 78 healthy participants in two age-groups: 40 participants (19 men, 21 women) in a younger cohort (mean age 27 years, standard deviation 4.4) and 38 participants in a mature cohort (22 women, 16 men; mean age 70 years, standard deviation 6.8). Each participant attended an intake appointment in which they completed a brief personal data form, documenting medical history and medication use to confirm eligibility, and provided informed consent for the experiment. Participants were accepted into the study, provided that they reported no prior history of swallowing, motor speech, gastro-esophageal or neurological difficulties, Type I Diabetes, chronic sinusitis, or taste disturbance. Individuals with a history of surgery to the speech or swallowing apparatus (other than routine tonsillectomy, adenoidectomy or minor dental surgery) were excluded. Individuals with full upper palate dentures were included, provided they were willing to remove their dentures for the experiment. Smokers (and those who had been smokers in the past year) were excluded, due to the possibility that smoking may alter oral sensory function [25, 26]. There were no exclusions on the basis of race or ethnicity.

### Stimuli

Thickened liquid stimuli were supplied for the project by Flavour Creations Inc., an Australian company that specializes in the production of xanthan gum-thickened liquids for use in dysphagia management. The data describe swallowing with three levels of thickened liquid, prepared by mixing xanthan gum into flavored cordials with thickener concentrations of 0.5, 0.63, and 0.87 w/w%, respectively. The cordials were comprised of water with either lime, raspberry, diet raspberry, or cranberry flavoring added to a fixed sucrose or sweetener concentration. Rheological testing showed that these stimuli had apparent viscosities of 190, 250, and 380 mPa s at 50/s at the recommended serving temperature of 10 °C, falling between the mildly thick and moderately thick levels of commercially available thickened liquids manufactured by Flavour Creations for clinical use. Note that the term apparent viscosity is used to describe the measurement of viscosity at a particular shear rate for liquids with non-Newtonian flow properties. Apparent viscosity did not differ significantly across the four flavors. Additional details regarding the rheological testing, together with evidence from an oral sensory discrimination experiment, showing that the different concentrations of xanthan gum were perceivably different have been reported elsewhere [27]. For reference, it can be noted that these measurements placed the stimuli in the upper nectar-thick to low honey-thick range according to the National Dysphagia Diet, the current taxonomy used for dysphagia thickened liquids in North America [28].

### Tongue Pressure Measurement

**Fig. 1:**
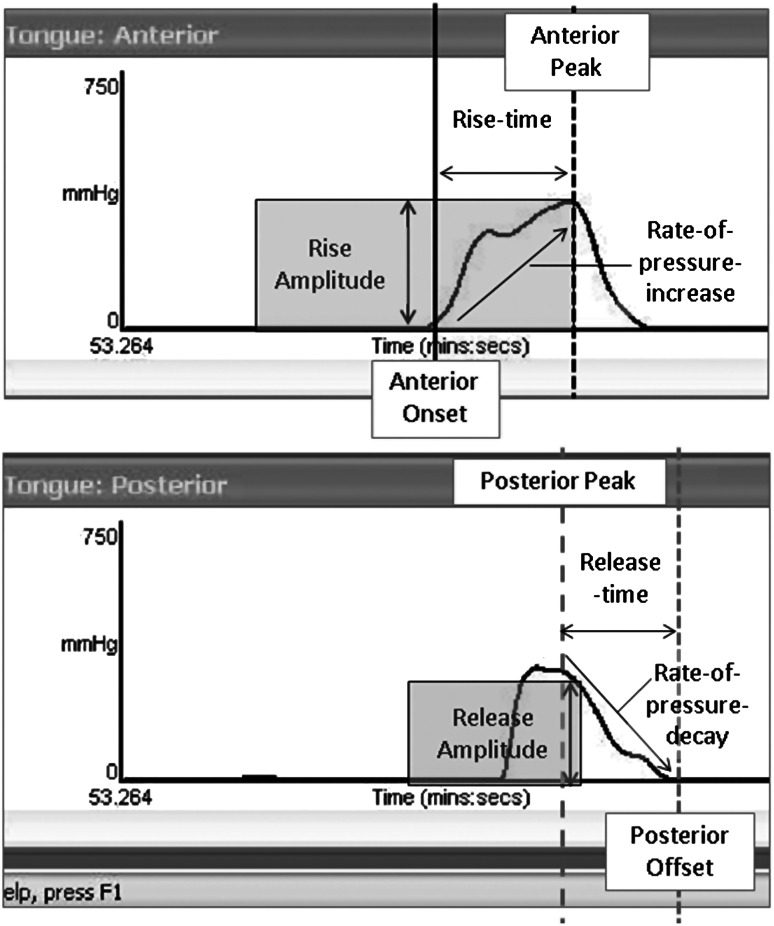
Tongue pressure waveforms with parameters marked. For the analysis, amplitude was defined as the greater of either rise or release amplitude (i.e., peak minus onset or offset amplitude), in mmHg. The rate-of-pressure-increase was calculated as pressure amplitude divided by rise-time, while the rate-of-pressure-decay was calculated as pressure amplitude divided by release-time

Tongue pressure data were collected using the lingual manometry module of the KayPentax Swallowing Signals Lab. Data were collected for two reference tasks (maximum isometric tongue-palate pressure generation and water swallows) and for swallows of the thickened liquid stimuli. Continuous measurements of pressure were registered at the anterior, mid, and posterior hard palate (up to 750 mmHg, at 250 Hz) using a soft silicon strip housing three pressure bulbs (13 mm diameter, 8 mm spacing). The pressure bulb strip was adhered to the participant’s palate in midline using a moldable double-sided Stomahesive^®^ strip (Convatec, St. Laurent, Quebec). Participants performed the experimental swallowing tasks in blocks of four repeated sips, with the order of blocks randomized. Each participant was randomly assigned to a single flavor of thickened liquid (lime, raspberry, diet raspberry, or cranberry); liquids were maintained at recommended serving temperature (10 °C) until just prior to use. Sip size was not strictly controlled; rather, participants were instructed to take each series of four naturally-sized sips from a single cup containing 60 ml, removing the cup from the lips between sips. Post-processing of the tongue pressure waveform data was done in Matlab using a segmentation algorithm, which identified departures from baseline, peak pressures and returns to baseline based on a moving window standard deviation function. The following parameters were derived for each pressure event (see Fig. 1 for an illustration):

- amplitude, defined as the difference between the largest peak and lowest rest pressure amplitudes recorded across the three sensors;
- rate-of-pressure-increase, defined as amplitude divided by rise-time; and
- rate-of-pressure-decay, defined as amplitude divided by decay-time.

### Analysis

The analysis began with a description of the tongue-palate pressure patterns seen during the reference task of water swallows. Pressure amplitudes were calculated in mmHg and also in values normalized to each participant’s maximum isometric pressure values (% maximum isometric pressure). The rates-of-pressure-increase and -decay were computed using normalized amplitude values (% maximum isometric pressure/second). Descriptive statistics for the thickened liquid swallows were calculated in units normalized to each participant’s water swallow parameters (% of water swallow value). A stepwise approach was used to build the statistical model for our research question, beginning with univariate tests for sex and age-group and flavor differences in the three dependent tongue pressure variables of interest (amplitude, rate-of-pressure-increase and rate-of-pressure-decay). No significant differences in any of these three parameters were found across stimulus flavor, justifying exclusion of this factor from subsequent statistical analyses. The amplitude parameter was found to vary according to sex and age-group. Consequently, the statistical model for exploring variations in tongue-palate pressure amplitudes was designed as a fully factorial linear mixed model repeated measures analysis of variance with factors of stimulus (190, 250 and 380 mPa s), age-group, and sex and a repeated factor of token-within-task. Given the absence of univariate effects of sex and age-group on the rates-of-pressure-change, a simpler model with a main factor of stimulus and a repeated factor of token-within-task was used for these parameters. A compound symmetry covariance structure was used based on demonstration of the best model fit using restricted log likelihood estimation. Separate analyses were performed for each dependent variable in SPSS version 22.0. An a priori alpha criterion was established at *p* < 0.05. Significant differences were further explored using Sidak tests for pairwise comparisons. Effect sizes for significant pairwise contrasts were calculated using Cohen’s *d*, for which values <0.5 are considered small, values between 0.5 and 0.8 are considered medium, and values >0.8 are considered large [29].

## Results

### Reference Tasks

Mean values for maximum isometric pressure amplitudes were measured at 460 mmHg [95 % confidence interval (CI) 451–468 mmHg] in the younger participants, and 376 mmHg (95 % CI 367–386 mmHg) in the mature cohort [*F*(1, 74.3) = 9.824, *p* = 0.002, *d* = 0.7, i.e., large effect size]. Water swallow amplitudes averaged 113 mmHg (95 % CI 109–116 mmHg), corresponding to 28 % of the maximum isometric pressure range (95 % CI 27–29 %). Water swallow amplitudes did not differ significantly between young and mature participants, either in mmHg or when expressed in normalized values.

### Amplitude

**Fig. 2:**
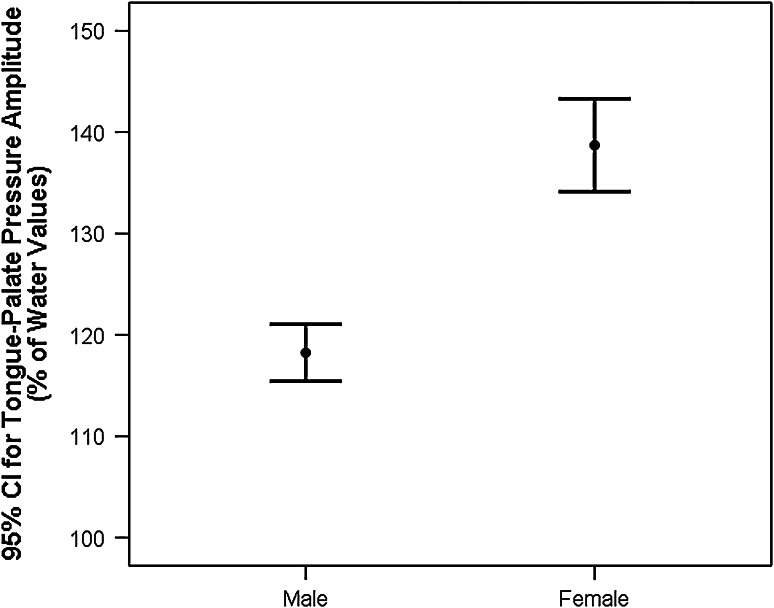
Sex differences in tongue-palate pressure amplitudes for thickened liquids. Female participants were found to use significantly higher tongue-palate pressure amplitudes than men when swallowing thickened liquids (*p* < 0.05). Values are expressed as a percentage of pressure amplitudes seen during a reference task of water swallows

**Fig. 3:**
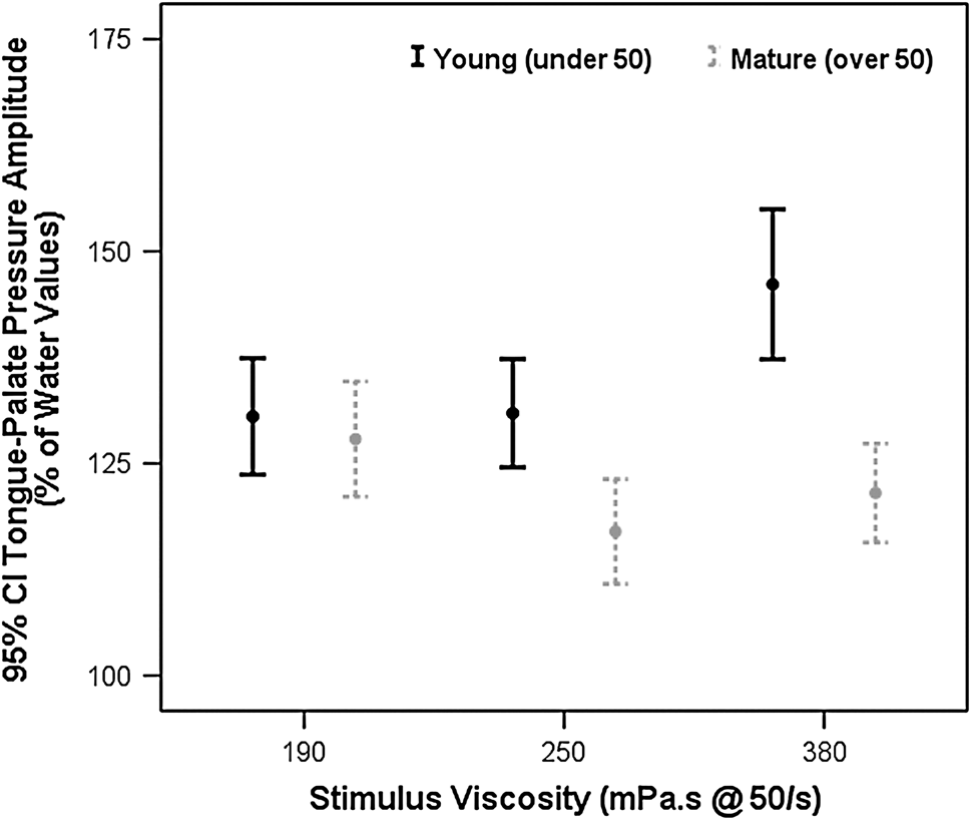
Stimulus and age-group differences in tongue-palate pressure amplitudes for thickened liquids. A significant stimulus × age-group interaction was found, in which younger participants used significantly higher tongue-palate pressure amplitudes than mature participants when swallowing thickened liquids (*p* < 0.05). Amplitudes for the 380-mPa s stimulus were significantly higher (*p* < 0.05) than for the two thinner liquids in the younger cohort

Overall, the 95 % CIs for swallowing pressures with the three xanthan gum-thickened liquids in this experiment ranged from 122 % to 134 % of the values seen during water swallows, reflecting greater amplitudes when swallowing thicker liquids. A significant main effect of sex was seen, [*F*(1, 73.25) = 20.4, *p* = 0.002, *d* = 0.48, i.e., small effect size], with female participants increasing the pressures used relative to water by a greater degree than male participants (see Fig. 2). Additionally, a significant stimulus × age-group interaction was found, [*F*(2, 825.54) = 7.57, *p* = 0.001], along with statistically significant main effects of stimulus, [*F*(2, 835.54) = 6.2, *p* = 0.002, *d* = 0.23, i.e., small effect size], and age-group, [*F*(1, 73.25) = 5.15, *p* = 0.03, *d* = 0.32, i.e., small effect size]. As shown in Fig. 3, the young participant cohort used a higher magnitude of pressure than the older participants on the 250 and 380 mPa s liquids, and they increased swallowing pressure amplitudes significantly for the thickest liquid at 145 % of values seen during water swallows (95 % CI 136–155 %). By contrast, the older participants displayed the greatest increase in swallowing pressure amplitudes relative to water for the 190 mPa s stimulus (126 % of water swallow values, 95 % CI 116–137 %), while pressures seen for the two thicker stimuli were lower, with the 95 % CIs spanning 105–131 % of water swallow values.

### Rates-of-Pressure-Change

**Fig. 4:**
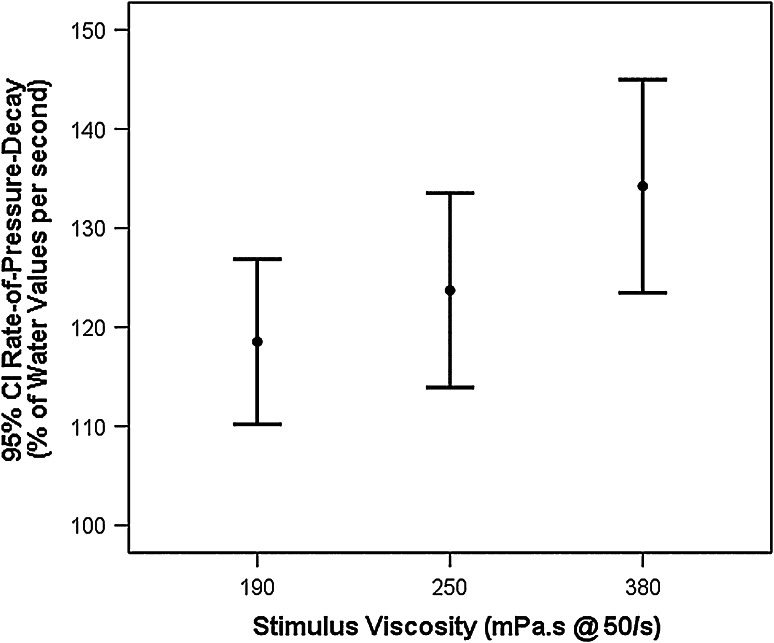
Stimulus differences in tongue-palate pressure decay for thickened liquids. A pattern of more rapid pressure decay for thicker liquids was seen, with significantly faster pressure decay (*p* < 0.05) for the 380 mPa s stimulus compared to the 190 mPa s liquid

The rate-of-pressure-increase for swallows of the three-thickened liquids showed no significant difference across stimuli, averaging 117 % of the values seen for water swallows (95 % CI 105–129 %). This contrasted with a significant main effect of stimulus for the rate-of-pressure-decay, [*F*(2, 807.1) = 3.2, *p* = 0.041, *d* = 0.19, i.e., small effect size], in which lower values of pressure decay (i.e., more gradual) were seen for the 190 mPa s liquid compared to the 380 mPa s liquid (see Fig. 4). Rates-of-pressure-decay increased gradually with increasing viscosity, with the combined 95 % CIs spanning 107–146 % of the values seen during water swallows.

## Discussion

These data demonstrate that healthy adults recruit higher amplitudes of tongue-palate pressure when swallowing nectar- and honey-thick xanthan gum-thickened liquids ranging from 190 to 380 mPa s, compared to the pressures used when swallowing water. Additionally, the temporal profile of tongue-palate pressures changes with increasing viscosity. A pattern of more rapid pressure decay is seen as liquids become thicker.

A number of limitations must be recognized as caveats to the observed results. First, the possibility that the presence of a sweet flavor in the thickened liquid stimuli might have contributed to the observed pressure differences compared to water cannot be ruled out. Second, sip volume was not strictly controlled, and the influence of sip size on tongue pressures remains unknown. Third, it was necessary for participants with full upper plate dentures to remove these for the experiment, to avoid damaging the dentures with the glue used to attach the pressure sensors. The impact of denture removal on swallowing is unknown. Finally, it is beyond the scope of this manuscript to speculate regarding the possible similarities or differences that might exist for liquids of equivalent apparent viscosities thickened with different thickening agents. The present manuscript reports novel data for xanthan gum-thickened liquids, which are available on an increasing basis in the clinical market, but we did not collect data for starch-thickened liquids of comparable viscosity; as such, caution is warranted in extrapolating from these results to liquids thickened with other thickeners.

The data in this study concur with previous studies in demonstrating decreased amplitudes of maximum isometric tongue-palate pressure in healthy seniors compared to younger individuals, with the lower 95 % CI falling at 367 mmHg (approximately 49 kPa). Consistent with previous findings [20], the present data also suggest that despite age-related reductions in maximum isometric tongue pressures, the pressure amplitudes used habitually in water swallowing tasks do not decline with age [30, 31].The data also concur with previous studies in demonstrating that the tongue-palate pressures used in water swallowing fall well below 30 % of maximum isometric task values. The amplitudes of pressure seen when swallowing the xanthan gum-thickened liquid stimuli in this experiment ranged from 122 to 134 % of the values seen when swallowing water. These values still fell at or below 40 % of maximum isometric pressure capacity in our healthy adult participants. These findings suggest that the presentation of nectar- and honey-thick stimuli thickened with xanthan gum is unlikely to tax tongue-palate pressure generation capacity, even in seniors with reduced maximum isometric tongue pressure measures.

Previously, it has been suggested that the rate-of-pressure-decay (also called “release-slope”) might reflect the degree to which the flow of a liquid bolus is being actively controlled by the tongue as it enters the pharynx [15]. More rapid withdrawal of pressure has been suggested as a mechanism that might contribute to more rapid flow of the bolus (or conversely, that more gradual withdrawal of pressure might slow bolus flow). In the case of the present data, thicker liquids recruited both higher amplitudes of pressure and more rapid rates-of-pressure-decay, consistent with the previously reported observations of this phenomenon. However, the clinical significance of this finding cannot be confirmed without direct visualization of bolus flow under videofluoroscopy.

The stimuli selected for this experiment had apparent viscosities of 190, 250, 380 mPa s at a shear rate of 50/s. Although the rheological spacing between these stimuli was not even in a linear sense, it should be recognized that the perception of increasing viscosity grows in an exponential fashion at approximately 1/5th the rate of the actual increase [32]. All three of the stimuli used in this experiment had previously been confirmed to be perceivably different from one another during oral sensory appraisal of liquid flow characteristics [27]. The data show that all three liquids elicited greater amplitudes of tongue-palate pressure than water. However, despite having perceivably different viscosities, pairwise comparisons showed that the significant differences lay between the thickest liquid and the two thinner stimuli (i.e., 380 vs. 250 and 190 mPa s). This finding implies that reasonably large differences in liquid viscosity within the nectar- and honey-thick ranges may be required to elicit differences in swallowing behavior. Certainly, viscosity differences that are narrower than perceptual discrimination thresholds seem unlikely to be large enough to alter swallowing. A question for future research will be to determine whether viscosity differences of the magnitudes tested in this study are perceivable and also elicit meaningful differences in tongue-palate pressure parameters in individuals with dysphagia. Furthermore, explorations using xanthan gum-thickened liquids with viscosities that are both thinner and thicker than those studied in this experiment are warranted to generate a more complete understanding of the increments of viscosity that may have clinical relevance in dysphagia management.

## Conclusions

This study confirms that xanthan gum-thickened liquids elicit alterations in both the amplitude and release characteristics of tongue-palate pressures that are applied during swallowing. Although thicker liquids (up to 380 mPa s) elicit higher amplitudes of tongue-palate pressure compared to water, the observed values should still be easily achieved by most adults, falling below 40 % of maximum isometric pressure values. Despite this fact, evidence that higher tongue pressures are typically used to propel thicker liquids through the oropharynx provides justification for treatment targeting improved tongue strength in individuals with reduced maximum isometric pressure capacity [33–37]. Similarly, this finding raises the need for caution when selecting thickened liquids for individuals with reduced tongue strength, and suggests that there may be a viscosity threshold that becomes too thick for effective oral processing in such cases. The data also suggest that a strategy of manipulating the rate of pressure release (i.e., more rapid release of higher amplitude tongue-palate pressures) may be in use during the swallowing of thicker liquids by healthy adults. This same finding could also be interpreted as evidence of the opposite pattern, namely a strategy of more gradual release of tongue-palate pressures during thin liquid swallows in healthy adults. Further explorations using direct observation of bolus flow under videofluoroscopy will be needed to confirm the clinical significance of this phenomenon. If a relationship between the rate-of-pressure-decay and bolus flow can be demonstrated, this may justify the addition of temporal goals to tongue-palate pressure treatment tasks.
